# The motivational foundations of childbearing: Investigating relationships between childbearing attitudes, value preferences, and parental bonding

**DOI:** 10.1371/journal.pone.0324243

**Published:** 2025-05-29

**Authors:** Eugene Tartakovsky, Mor Mizrahi

**Affiliations:** The School of Social Work, Tel-Aviv University; McGill University Faculty of Arts, CANADA

## Abstract

This study aims to reveal the motivational foundations of childbearing. To achieve this goal, we investigated how general motivational goals expressed in personal value preferences and relationships with one’s parents are connected to attitudes toward childbearing. The study is based on Schwartz’s theory of values [1]. The study was conducted in Israel among young adults (age 18–35) with no children (n = 1122). Higher preferences for tradition and benevolence-care and lower preferences for self-direction and universalism-nature values were associated with positive childbearing attitudes among men and women. In addition, higher preferences for security-social values were associated with positive childbearing attitudes among men. Maternal bonding was associated with positive childbearing attitudes among men and women, and paternal bonding was associated with positive childbearing attitudes among men. High religiosity, high income, and low education were associated with positive childbearing attitudes among women. Among men, only the effect of religiosity was significant. No difference between men and women in childbearing attitudes was found. Personal value preferences partly mediated the effects of parental bonding and socio-demographic variables on childbearing attitudes. The obtained results advance our understanding of the psychological mechanisms of childbearing.

## Introduction

During the last decades, we have witnessed a global decrease in childbearing. From 1963 to 2023, the average number of children per woman decreased worldwide from 5.3 to 2.4, and this trend continues [2]. Researchers connect the decrease in childbearing to economic growth, an increase in education, a decrease in religiosity, and a rise in women’s rights, employment, and social status [3]. It has also been connected to growing fears of natural resource depletion, increased greenhouse gas emissions, and food insecurity due to overpopulation [4].

The motivational mechanisms of childbearing have been rarely investigated, and the present study aims to narrow this gap. In this study, we focus on childbearing attitudes. Attitudes refer to emotions and beliefs toward a particular object, person, thing, or event. Attitudes can also be described as the evaluations (positive or negative) of something or someone [5]. Thus, childbearing attitudes reflect how much an individual considers childbearing to be positive or negative, desirable or not, and whether it brings joy and happiness or hardships and distress [6–8]. Numerous studies have established that attitudes affect behavior [9,10], including childbearing [6]. Therefore, we should identify the factors affecting childbearing attitudes to understand why people bring or do not bring children into the world.

Our study is based on Schwartz’s theory of values, the most comprehensive and empirically sound theory of human motivations [1]. Previous studies have confirmed the values-attitudes link and demonstrated that people have positive attitudes toward a behavior when it contributes to attaining their preferred motivational goals as expressed in personal value preferences [11–15]. However, this theoretical model has not been applied to childbearing.

The present study’s primary goal is to reveal the motivational foundations of childbearing, i.e., to understand the attainment of which motivational goals is promoted or impeded by childbearing. To achieve this goal, we investigated the connections between general motivational goals expressed in personal value preferences and childbearing attitudes. In addition, we investigated the effect of relationships with parents on childbearing attitudes. Previous studies have established the direct connection between relationships with parents and childbearing [16]. The present study advances the existing knowledge by investigating the possibility that the relationships with parents indirectly affect childbearing attitudes by influencing the individual’s motivational goals expressed in personal value preferences.

We conducted our study in Israel, the country with the highest childbirth rate among developed countries (2.94) [17]. Israel upholds the high birthrate by numerous means, e.g., promoting the pronatalist ideology, offering numerous free IVF, and providing a high level of free perinatal medical care [18–22]. The high birthrate in Israel has important political implications, considering the Arab-Jewish conflict and tension between Orthodox and non-Orthodox Jews [20,23,24]. Previous studies have shown that when society encourages a behavior, the link between attitudes and behavior is strengthened [6,10]. Therefore, childbearing attitudes should strongly impact childbearing behavior in Israel, increasing the practical importance of understanding factors affecting childbearing attitudes.

As has been done in some previous studies [25–27], to investigate childbearing attitudes, this research focused on people who have no children. This decision was based on the idea that personal characteristics and other precursors of behaviors (such as personal value preferences) should be studied before initiating the behavior [13,28]. If we asked people with children about their attitudes toward childbearing, they would provide a post hoc explanation for a behavior already occurring. Thus, their responses would reflect their experience with their children. In addition, their responses would be affected by their explanatory style and the rationalization tendency.

### Factors affecting childbearing

A systematic literature review [8] has demonstrated that positive childbearing attitudes are associated with advanced age, higher education, stable jobs, a better financial situation, and marriage among young adults. At the same time, the importance of obtaining an education and developing a career has been associated with delays in childbirth and less positive childbearing attitudes [6]. Religiosity and the traditional way of life were positively associated, and the importance of self-realization was negatively associated with childbearing attitudes [7,8]. Previous studies have also demonstrated that education, career, and economic independence have a stronger effect on childbearing attitudes among women than men [29]. However, researchers note that men’s motivation to enter parenthood is studied less than women’s motivation [8].

Researchers investigating the psychological aspects of religion in childbearing note that religion provides ideological incentives to bear children and seeks to regulate reproductive behavior and promote fertility [30,31]. On the other hand, people committed to autonomy, self-expression, and individual control over their bodies, sexuality, and intimate relationships are less religious and less inclined to childbearing [7,30].

Scholars who connect childbearing attitudes to children’s values note that children provide intergenerational continuity, symbolic personal immortality, and social support for older parents, i.e., they promote the attainment of collectivistic motivational goals. On the other hand, individualistic motivations, such as self-actualization (e.g., education and career) and hedonism (e.g., luxury spending) have been considered to contradict childbearing because children deplete parents’ time, money, and energy resources [6,32]. Thus, collectivist and individualist motivations contradict each other in their effect on childbearing.

### Theory of human values

Shalom Schwartz’s [1] theory of human values defines values as desirable trans-situational goals guiding people’s lives. Value preferences reflect the individuals’ general motivational goals, which affect the perception of reality and direct behavior [13]. The theory specifies a comprehensive set of 19 motivationally distinct values: power (dominance and resources), achievement, hedonism, stimulation, self-direction (thought and action), universalism (concern, nature, and tolerance), benevolence (caring and dependability), humility, conformity (rules and interpersonal), tradition, security (personal and social), and face [33].

The theory assumes the existence of dynamic relations between the values in that the pursuit of each value has consequences that may conflict or may be congruent with the pursuit of other values [33]. The theory specifies four higher-order values and defines the relationships among them as complementary or conflictual. Openness to change values (including self-direction and stimulation) emphasizes readiness for new ideas, actions, and experiences. They contrast with conservation values (including conformity, tradition, and security) that emphasize self-restriction, order, and preservation of the status quo. Self-enhancement values (including power and achievement) emphasize pursuing one’s interests. They contrast with self-transcendence values (including universalism and benevolence) that emphasize the transcendence of one’s interests for the sake of others.

The theory further assumes that the values of openness to change and self-enhancement have a personal focus, i.e., they are concerned with outcomes for the self. The values of conservation and self-transcendence have a social focus, i.e., they are concerned with outcomes for others or established institutions. Finally, the theory assumes that the values of self-transcendence and openness to change express the motivations directed toward psychological growth and development. They are likely to motivate people when they are relatively free of anxiety. The values of conservation and self-enhancement are directed toward protecting the self against anxiety and threats [33].

### Values and childbearing

As mentioned above, values reflect general motivational goals [1]. Therefore, the values theory may enable us to understand the motivational goals of childbearing. Specifically, revealing the pattern of connections between personal value preferences and childbearing attitudes will let us understand which general motivational goals are promoted and which are impeded by childbearing. We found no research on the connections between personal value preferences and childbearing attitudes. Therefore, to formulate the hypotheses on the values-childbearing attitudes connection, we used the results of studies on the connections of socio-demographic variables and collectivism-individualism motivations with childbearing attitudes reviewed above, on the one hand, and studies on the connection between these variables and values, on the other hand.

The most consistent finding of previous studies is the link between religiosity and childbearing [30,31]. Since religiosity is strongly connected to tradition values [33], we hypothesize that a higher preference for tradition values is associated with positive childbearing attitudes (**H1**). On the other hand, self-actualization through pursuing education and a career has been associated with negative childbearing attitudes [6,32]. Since self-actualization is associated with self-direction values [33], we hypothesize that a higher preference for these values is associated with negative childbearing attitudes (**H2**).

Raising children is costly and may deplete parents’ time, money, and energy resources [6,32]. In Schwartz’s theory, resources are associated with power values [1]. Therefore, we hypothesize that a higher preference for power values is associated with negative childbearing attitudes (**H3**). On the other hand, raising children requires love and care [6], and care for close others is the main motivational goal of benevolence values [33]. Therefore, we hypothesize that a high preference for benevolence values is associated with positive childbearing attitudes (**H4**).

Finally, fears of overpopulation, depletion of natural resources, and climate problems have been associated with demands to decrease human fertility [4]. Protecting nature is the main motivational goal of universalism-nature values [33]. Therefore, we hypothesize that a higher preference for these values is associated with negative childbearing attitudes (**H5**).

### Relationships with parents and childbearing

Most studies on the effect of relationships with parents on childbearing have been based on the attachment theory [16]. It is not surprising, given that the attachment theory is widely used to explain the effect of parenting on individual development and functioning [34]. Most studies found that individuals who experienced positive parenting and developed a secure attachment style reported more positive attitudes toward childbearing. Specifically, they reported a stronger desire to have children and to have a larger number of children, plans for an earlier birth, the perception of parenting as less threatening and concerning, more positive expectations regarding the stress of childrearing, and more positive expectations to succeed as parents. However, the effects of insecure attachment styles varied across studies: most found that only avoidant attachment was associated with negative childbearing attitudes and behavior. In contrast, findings regarding the negative effect of anxious style were inconsistent [16].

Despite the importance of previous studies on the effect of attachment on childbearing, they have several drawbacks. First, most of these studies focused on attachment styles in relationships with peers, not parents. Second, attachment styles imprecisely reflect the relationships with parents because other people also affect the individuals’ attachment styles. Finally, the attachment theory assumes that relationships with parents are directly copied onto other relationships [35], and it does not consider alternative transmission mechanisms.

The present study advances the existing knowledge on the effect of parenting on childbearing in several directions. First, we measured the individuals’ perception of their parents’ parenting behavior, not attachment styles. We applied Parker’s concept of parental bonding, which considers parenting to vary along two dimensions: care vs. neglect and autonomy-providing vs. over-control [36]. Previous studies have demonstrated that caring and autonomy-providing parenting leads to the development of a secure attachment style, while neglect and over-control lead to insecure attachment [37,38]. Therefore, we hypothesize that positive parental bonding (care and autonomy-providing) is associated with positive childbearing attitudes (**H6**).

Second, we measured maternal and paternal bonding separately, which permitted us to differentiate between the effects of relationships with mother and father on childbearing attitudes. An old tradition in psychology assumes that relationships with the mother have a stronger impact on a child’s development than relationships with the father [39]. Therefore, in the present study, we hypothesize that maternal bonding has a stronger effect on childbearing attitudes than paternal bonding (**H7**).

Finally, as mentioned above, attachment theory assumes the existence of a modeling mechanism when individuals copy their experience with their parents onto other interpersonal relationships [16,40]. The present study assumes the existence of an additional mechanism connecting parenting with childbearing attitudes through parenting’s influence on values. Specifically, we assume that parenting affects the development of individuals’ general motivational goals. In turn, general motivational goals affect the individuals’ childbearing attitudes. General motivational goals find their expression in value preferences [1]. Thus, personal value preferences may partly mediate the connection between one’s experience with parents and childbearing attitudes (**H8**).

Previous studies have investigated the connection between attachment styles and personal value preferences. These studies found that the secure attachment style was associated with a higher preference for self-transcendence values, and the avoidance attachment style was associated with a lower preference for self-transcendence values [40]. In addition, securely attached individuals reported higher preferences for openness to change values, whereas insecure individuals had higher preferences for conservation values [41]. Following previous studies on attachment and values, we hypothesize that positive parental bonding is associated with high preferences for self-direction, universalism, and benevolence values and low preferences for power, security, tradition, and conformity values (**H9**).

## Method

### Participants

This study used a representative sample of young Israelis with no children (n = 1122). We limited our sample to non-ultraorthodox Jews and did not include Palestinian Israelis and ultraorthodox Jews in the study because these groups have distinctive patterns of fertility, family structure, and value preferences [14,21,42,43]. The sample comprised 52.7% females, 47% males, and.03% others. The sample’s mean age was 26.2 (*SD* = 4.62, range = 18–35). 64% of the participants had a post-secondary education. 45% defined themselves as secular (i.e., did not follow religious practices), 32% were traditional (i.e., followed some religious practices), and 23% were religious (i.e., strictly followed religious practices). These characteristics are similar to those reported for young Jewish Israelis [44].

### Procedure

The Tel Aviv University Review Board approved the study (IRB approval number 0006988–1/ 20.7.23). The study used a representative sample of the non-ultraorthodox Jewish Israeli population stratified by gender, religiosity, and education. Two research companies have conducted the survey under the researchers’ supervision. Individuals aged 18–35 who did not have a child were invited to participate in the study. The questionnaires were in Hebrew. The questionnaires were distributed using Qualtrix. The participants received a standard compensation of about $5. Participation in the study was voluntary and anonymous. All participants signed a written informed consent form. The study was conducted in September 2023. The research was partly funded by the Israel Science Foundation Research Grant (218/22).

### Instruments

#### Childbearing attitudes.

A 10-item scale (five positive and five negative items) was created for this study to measure childbearing attitudes. The items were adapted from the literature [6,32,45]. The respondents reported their agreement with the items on a 6-point scale, from 1 – *fully disagree* to 6 – *fully agree*. Example items: “I am sure my life will improve when I have children.” “I fear it will be difficult for me when I have children.” The negative items were upended, and the mean of the ten items was used as the scale score. Higher scores mean a more positive attitude toward childbearing. The scale demonstrated high internal consistency (Cronbach’s *α* = .89). To test the scale’s validity, we correlated it with the question “Do you want to have children?” (3.5% said no, 6.9% were not sure, and 89.7% said yes) and with the desired number of children (*M(SD)* = 3.51(2.03)). The obtained high positive correlations (*r* = .63,.57) corroborated the scale’s validity. Finally, the childbearing attitudes scale was highly positively correlated (*r* = .54) with a care and tenderness scale measuring attitudes toward children in general [46], again corroborating the scale’s validity.

#### Personal value preferences.

Personal value preferences were measured using the 57-item Portrait Values Questionnaire-Revised (PVQ-R) [33]. Each item portrays an abstract person describing their goals, aspirations, and wishes that indicate the importance of a specific value. Respondents indicate the described person’s similarity to them on a 6-point scale, from 1 – *not like me at all* to 6 – *very much like me*. Item example: “It is important to him/her to avoid upsetting other people.” (Conformity-interpersonal). Internal consistency of all values was good (Cronbach’s alphas):.69-.89. As recommended [33], each participant’s responses were centered on their mean for all values to correct individual differences in using the response scales. Thus, the average of all 57 values was subtracted from each value score.

#### Parenting.

The individuals’ perception of their parents’ parenting behavior was measured by the 25-item Parental Bonding Inventory (PBI) [37]. PBI describes parenting behavior along two dimensions: care-neglect and autonomy-overcontrol. Respondents are asked how often they experienced the specific parenting behaviors of their parents in their childhood and adolescence on a 6-point scale, from 1 – *never* to 6 – *always*. The questions were formulated separately for mothers and fathers. Item examples: “My father spoke to me warmly and friendly” (Care). “My mother let me decide things for myself.” (Autonomy). For both parents, the care-neglect and autonomy-overcontrol scales were highly correlated (*r* = .61,.62). Therefore, for each parent, we combined the two scales into one scale measuring maternal or paternal bonding by averaging the care-neglect and autonomy-overcontrol scales’ scores. Internal consistency of both scales was high (Cronbach’s alpha): *α* = .87,.92. Higher scores on the maternal and paternal bonding scales mean a more positive experience with mother and father.

### Data analysis

**Fig 1:**
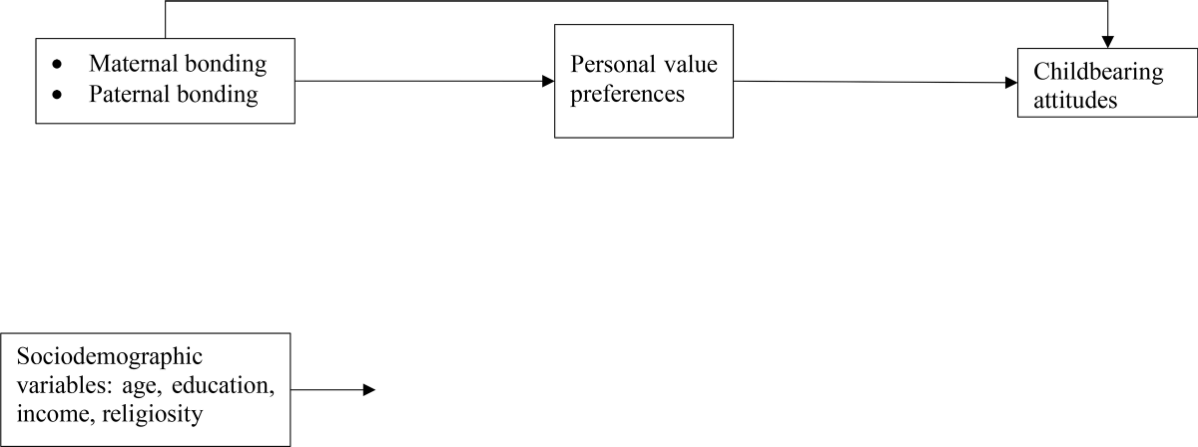
Research model.

First, we conducted pairwise analyses using SPSS 29 [47]. We calculated bivariate correlations between values and childbearing attitudes for men and women and compared childbearing attitudes of men and women using a Univariate Analysis of Variance (controlling for age, education, income, and religiosity). Second, we conducted a multigroup path analysis (for women and men) using Mplus 7 [48]. Fig 1 presents the hypothesized model that includes the following variables: maternal and paternal bonding, nine basic values significantly correlated with childbearing attitudes, childbearing attitudes, and five socio-demographic variables. To obtain a more parsimonious model, when sub-values (e.g., self-direction thought and self-direction action) were significantly correlated with childbearing attitudes, we included the combined value in the analysis (self-direction). When one of the sub-values was not significantly correlated with childbearing attitudes (e.g., security-social but not security-personal), we included only the significantly correlated sub-value in the model. After the initial test of the model, four values whose effect on childbearing attitudes in the path analysis was non-significant (universalism-concern, humility, power, and hedonism) were excluded, and the final path analysis was run with five remaining values (self-direction, security-social, tradition, benevolence-care, and universalism-nature). Sociodemographic variables (age, education, income, and religiosity) were connected to all other variables in the model. The direct and indirect effects were tested using the bootstrapping method with 1000 re-samples with a 95% confidence interval.

## Results

### Pairwise analyses

Table 1 presents correlations between basic values and childbearing attitudes separately for women and men. Among women and men, childbearing attitudes were positively correlated with tradition (*r* = .47, *p* < .001/ *r* = .45, *p* < .001) and benevolence-care values (*r* = .19, p < .001/ *r* = .11, *p* = .012), and negatively correlated with self-direction (*r* = -.29, *p* < .001/ *r* = -.11, *p* = .013) and universalism-nature values (*r* = -.32, *p* < .001/ *r* = -.30, *p* < .001). In addition, among women, childbearing attitudes were positively correlated with humility (*r* = .10, *p* = .021) and negatively correlated with universalism-concern values (*r* = -.10, *p* = .014). Among men, childbearing attitudes were positively correlated with security-social (*r* = .15, *p* < .001) and negatively correlated with power (*r* = -.14, *p* < .001) and hedonism values (*r* = -.12, *p* = .008). A Univariate Analysis of Variance controlling for age, education, income, and religiosity found no significant effect of gender on childbearing attitudes (*F*(2; 1110) = 1.76, *p* = .173).

**Table 1:** Values and childbearing attitudes: pearson correlation coefficients.

Values	Women	Men
Self-Direction	-.29^***^	-.11^*^
Self-Direction Thought	-.23^***^	-.12^*^
Self-Direction Action	-.23^***^	-.06
Stimulation	.08	-.02
Hedonism	-.04	-.12^**^
Achievement	.01	.07
Power	-.07	-.14^***^
Power Dominance	-.06	-.10^*^
Power Resources	-.07	-.14^**^
Face	-.01	-.06
Security	.02	.09^*^
Security Personal	-.01	-.03
Security-Social	.04	.15^***^
Conformity	.01	.03
Conformity Rules	.02	.04
Conformity Interpersonal	-.01	.01
Tradition	.47^***^	.45^***^
Humility	.10^*^	.01
Benevolence	.14^***^	.11^*^
Benevolence Care	.19^***^	.11^*^
Benevolence Dependability	.04	.07
Universalism	-.26^***^	-.22^***^
Universalism-Nature	-.32^***^	-.30^***^
Universalism Concern	-.10^*^	-.01
Universalism Tolerance	-.05	-.02

*Note*:

* *p* < .05,

** *p* < .01,

*** *p* < .001

### Path analysis

**Fig 2:**
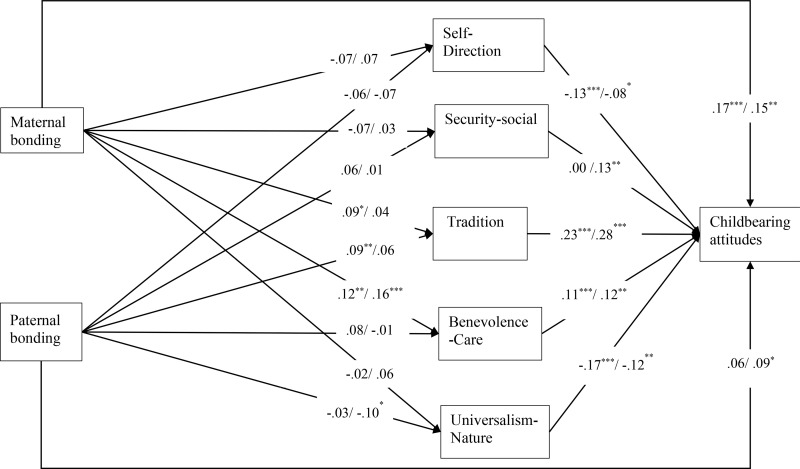
Path analysis: standardized direct estimates (Women/ Men).

The model was saturated and, therefore, demonstrated a perfect fit. The VIFs of all variables varied from 1.021 to 1.922, indicating no multicollinearity problems. The proportion of variance explained in childbearing attitudes was 35% for women and 33% for men. The path analysis’s standardized direct and indirect effects are presented in the S1 Appendix. Fig 2 presents standardized direct effects for women and men. To avoid cluttering, the figure does not include sociodemographic variables.

#### Direct effects.

Among women and men, tradition (*β* = .23, p < .001/ *β* = .28, p < .001) and benevolence-care (*β* = .11, *p* = .001/ *β* = .12, *p* = .003) were positively connected to childbearing attitudes. In addition, among women and men, self-direction (*β* = -.13, *p* < .001/ *β* = -.08, *p* = .036) and universalism-nature (*β* = -.17, *p* < .001/ *β* = -.12, *p* = .004) were negatively connected to childbearing attitudes. Finally, among men, security-social was positively connected to childbearing attitudes (*β* = .13, *p* = .001).

Maternal bonding was directly connected to positive childbearing attitudes among women and men (*β* = .17, *p* < .001/ *β* = .15, *p* = .001). Paternal bonding was also associated with more positive childbearing attitudes; however, the connection was significant only for men (*β* = .09, *p* = .033). Maternal bonding had a stronger effect on childbearing attitudes than paternal bonding for women (*t*(1178) = 2.18, *p* = .030) bu*t* not for men (*t*(1048) = 1.18, *p* = .239).

Several direct effects of sociodemographic variables on childbearing attitudes were significant. Among women, religiosity (*β* = .14, *p* = .001) and income (*β* = .10, *p* = .008) were positively, and education (*β* = -.10, *p* = .014) was negatively connected to childbearing attitudes. Among men, only religiosity was positively connected to childbearing attitudes (*β* = .16, *p* = .001).

Several effects of parental bonding on values were significant. Maternal bonding was positively connected to benevolence-care among women and men (*β* = .12, *p* = .008/ *β* = .16, *p* = .001) and tradition among women (*β* = .09, *p* = .010). Paternal bonding was positively connected to tradition among women (*β* = .09, *p* = .008) and negatively connected to universalism-nature among men (*β* = -.10, *p* = .042).

Several direct effects of sociodemographic variables on values were significant. Among women, education was negatively connected to tradition (*β* = -.10, *p* = .008); income was negatively connected to universalism-nature (*β* = -.09, *p* = .040); and religiosity was negatively connected to self-direction (*β* = -.17, *p* < .001), positively to tradition (*β* = .63, *p* < .001), and negatively to universalism-nature (*β* = -.21, *p* < .001). Among men, education was positively connected to self-direction (*β* = .15, *p* = .003) and negatively to tradition (*β* = -.08, *p* = .036); income was positively connected to security-social (*β* = .12, *p* = .019); and religion was positively connected to tradition (*β* = .57, *p* < .001) and negatively to universalism-nature (*β* = -.25, *p* < .001).

#### Indirect effects.

Positive indirect effects of parental bonding on childbearing attitudes were found among women for maternal bonding (*β* = .045, *p* = .008) and paternal bonding (*β* = .043, *p* = .013). Among men, all indirect effects of bonding on childbearing attitudes were non-significant. Several indirect effects of socio-demographic variables on childbearing attitudes through values were significant: religiosity among women (*β* = .230, *p* < .001) and men (*β* = .194, *p* < .001), and income (*β* = .065, *p* = .004) and education (*β* = -.051, *p* = .019) among men.

## Discussion

The present study tested a theoretical model connecting personal value preferences and parental bonding to childbearing attitudes. The study results corroborated the theoretical model and demonstrated that general motivational goals expressed in personal value preferences and relationships with parents conceptualized as parental bonding are directly connected to childbearing attitudes. In addition, parental bonding indirectly affected childbearing attitudes through its connection to values, albeit only among women.

### Value preferences and childbearing attitudes

Higher preferences for tradition and benevolence values and lower preferences for self-direction and universalism-nature values were associated with positive childbearing attitudes among women and men. In addition, higher preferences for security-social values were associated with positive childbearing attitudes among men. Considering the motivational goals of these values [33], the present study reveals which motivational goals are compatible with and which are contradictory to childbearing.

The motivational goal of tradition values is maintaining and preserving cultural, family, and religious traditions [33]. Childbearing may promote attaining these goals in several ways. First, bringing children into the world is an obligation in all religions. People who bear children fulfill the god’s commandment and, thus, preserve their religious tradition. Second, childbearing is a traditional behavior transmitted from generation to generation. Therefore, people who bear children confirm their adherence to the group’s tradition. Finally, by bringing children into the world, individuals ensure their place in the generational chain, thus maintaining the continuity of the group’s tradition and culture [30,31].

The motivational goal of benevolence-care values is to care for the welfare of close others, mostly family and in-group members [33]. This goal arises from the need for affiliation and promotion of the flourishing of one’s group [49]. Childbearing may promote the attainment of benevolence values’ motivational goals in two ways. First, it provides the individual with somebody to love and care for, thus fulfilling the affiliation needs. Second, childbearing increases the number of in-group members who may care for other in-group members.

Finally, a high preference for security-social values was connected to positive childbearing attitudes, albeit only among men. The motivational goal of these values is to preserve safety and stability in society. Thus, among men, childbearing is perceived as increasing the safety of one’s group. This is probably because larger groups are felt as stronger and more secure [50]. In addition, the group size may be associated with the military might [51]. This explanation of the connection between security-social and childbearing attitudes may be especially relevant in Israel due to the Arab-Israeli conflict and mandatory military conscription [52]. Interestingly, this connection was not found among women. It is possible that men and women ascribe social security to different factors and that women are more concerned with the dangers their children may be exposed to when defending society [53].

Benevolence-care, tradition, and security-social values have a social focus, i.e., concerned with outcomes for others and established institutions [33]. In other words, they are collectivist values [1,54]. The positive connections between these values and childbearing attitudes indicate that childbearing promotes the attainment of collectivist goals, strengthening the community in tangible and symbolic ways [54]. Thus, our findings corroborate previous studies on the collectivist nature of childbearing [21,22,54]. However, we have been able to provide a more detailed account of the motivational goals underlying childbearing.

Let us now consider which values are incompatible with childbearing. We found that a high preference for self-direction values was associated with negative childbearing attitudes. The motivational goals expressed in these values include the freedom to cultivate one’s ideas and abilities and determine one’s actions [33]. This connection indicates that individuals with a high preference for self-direction values perceive childbearing as limiting their freedom and preventing them from attaining their developmental goals. It is understandable given how much time, money, and energy childbearing requires [6,32]. These findings corroborate previous studies that found that self-actualization was negatively connected to childbearing [6,8,29].

In addition, a high preference for universalism-nature values was associated with negative childbearing attitudes. The motivational goals of these values include protecting and preserving the natural environment, which is derived from the survival needs that become apparent when people are aware of the scarcity of natural resources [49]. As mentioned above, childbearing is often connected to the fear of overpopulation associated with environmental damage [4]. Thus, individuals who care for the environment may have negative childbearing attitudes due to environmental concerns.

### The effect of parenting

In this study, we revealed the direct and indirect effects of parenting on childbearing attitudes. As hypothesized, maternal bonding was directly associated with positive childbearing attitudes among men and women; however, the effect of paternal bonding was significant only for men. Thus, the results of our study have corroborated previous findings indicating that relationships with parents directly affect childbearing attitudes through the copying mechanism [16,37–39]. We have also demonstrated that relationships with mothers stronger affect childbearing attitudes compared to relationships with fathers; however, the difference was significant only for women. The reasons for the differences in the effect of parenting on childbearing attitudes among women and men are unclear and require further research.

Besides, we found that both maternal and paternal bondings are indirectly connected to childbearing attitudes through their connections to values, albeit only among women. Specifically, maternal bonding was connected to childbearing attitudes through its positive connections with benevolence-care and tradition values. Thus, women with good relationships with their mothers consider caring for close others and preserving family traditions important motivational goals. Furthermore, they want children because childbearing permits them to care for a close other and continue the family. In addition, paternal bonding was connected to childbearing attitudes through its connection with tradition values. It means that women with good relationships with their fathers tend to preserve family traditions and consider achieving this goal through childbearing. It is unclear why the mechanism of indirect connection between parental bonding and childbearing attitudes is weaker among men. Further research is required to understand the difference between women and men in this regard.

### The effects of socio-demographic variables

We found no difference between men and women in childbearing attitudes. This finding contradicts “the common knowledge” that conceptualizes childbearing as “a maternal instinct” and, therefore, considers it more important for women than for men [18,55]. The equally positive childbearing attitudes among women and men may be a recent phenomenon related to changes in the gender hierarchy in modern society and stronger gender equality [56]. However, it may also be a specifically Israeli phenomenon resulting from the pronatalist nature of Israeli society that demands women and men to have children [18,20–22].

Among women, religiosity and income were directly positively connected to childbearing attitudes, while the connection with education was negative. Among men, only religiosity was positively connected to childbearing attitudes. These findings mostly corroborate the results of previous studies on socio-demographic variables and childbearing [6,8,29,30]. Different psychosocial mechanisms may underlay these findings. For instance, higher religiosity and lower education may be associated with pronatalist social norms [30]. In addition, resources associated with higher income may provide the material base for positive childbearing attitudes [29]. The positive effect of religiosity on childbearing attitudes may be especially strong in Israel because Israel is more religious than other developed countries [57], and previous studies have demonstrated the strong effect of religiosity on personal value preferences in Israel [14,43,58].

The present study found several indirect effects of socio-demographic variables on childbearing attitudes through personal value preferences. The strongest indirect effect among women and men was for religiosity. In both genders, religiosity positively affected childbearing attitudes through its positive connection with tradition and negative connection with universalism-nature values. In addition, among women, religiosity affected childbearing attitudes through its negative connection with self-direction values. These findings indicate that more religious people have more positive childbearing attitudes because they have a specific set of values, i.e., a higher preference for tradition and lower preferences for universalism-nature and self-direction (for women). These findings corroborate the results of previous studies regarding the connections between values and religiosity [33]. They also provide a psychological explanation for the connection between religiosity and childbearing found in previous sociological studies [30–31].

Among men, education had a negative effect on childbearing attitudes through its negative connection with tradition values. Less educated men have a higher preference for tradition values and, therefore, more positive childbearing attitudes. This finding corroborates previously found negative connections between education and tradition values [33] and indicates that less educated men have more positive attitudes towards childbearing because they have more traditional values. Surprisingly, the indirect effect of education on childbearing attitudes was not found among women. It may be explained by the changing status of women in higher education, where they constitute the majority (60% of Israeli students are women), and their numbers continue to grow [59]. Thus, higher education for women has become normative and less related to their values than in the past.

Finally, income was positively connected to childbearing attitudes among men through its positive connection to security-social values. This finding indicates that more affluent men may have more positive childbearing attitudes because they consider children a means of strengthening the security of their ingroup. The fact that this connection was not found among women indicates a different meaning of children for men and women in the context of wealth and social security [18].

### Limitations and suggestions for further research

Several study limitations must be considered. First, the study was cross-sectional; therefore, causal inferences cannot be drawn from the results. In addition, there might be a ‘recall bias,’ i.e., the participants’ current conditions might shape how they recall relationships with their parents in childhood and adolescence. Future longitudinal research would represent a significant advancement in the current findings. The second limitation of the present study relates to the research population. The suggested theoretical model was tested in only one ethnic group. Testing the model in other groups and countries is essential to its generalization. The third limitation of the study relates to its focus on individual-level factors and not investigating the mezzo- and macro-level factors that might afect childbearing attitudes. For instance, peers and mass media may influence childbearing attitudes. Finally, our sample was limited to young adults without children. Further studies should include other age groups and individuals already having children.

### Conclusions

The present study advances our knowledge of childbearing attitudes in several ways. First, it reveals the motivational goals of childbearing. We demonstrated that the motivational goals of preserving tradition and caring for close others, reflected in tradition and benevolence-care values, are compatible with childbearing. On the other hand, the goals of protecting the freedom to cultivate one’s ideas and abilities, determine one’s actions, and preserve the natural environment reflected in self-direction and universalism-nature values interfere with childbearing in both genders. In addition, among men, childbearing is perceived as compatible with maintaining safety and stability in society, the motivational goals expressed in security-social values.

Second, in the present study, we investigated the connection between childbearing attitudes and the individual’s relationships with their parents and revealed two mechanisms underlying this connection. The copying mechanism underlies the direct influence of parenting on childbearing when positive parenting directly leads to positive childbearing attitudes. Another mechanism underlies the indirect influence of parenting on childbearing through values. Here, positive parenting affects childbearing attitudes by forming higher preferences for the general motivational goals reflected in benevolence-care and tradition values. The discovery of these mechanisms advances our understanding of the effect of parenting on value preferences and childbearing.

Finally, the present study allowed us to better understand the connections between socio-demographic variables and childbearing. In addition to corroborating previous findings on direct connections between childbearing and socio-demographic variables (religiosity, education, and income), we revealed indirect effects of socio-demographic variables on childbearing through value preferences. These findings allow us to explain the socio-demographic differences in childbearing through the variations in general motivational goals across socio-demographic groups. The present study findings advance our understanding of childbearing’s motivational mechanisms and explain why individuals from different socio-demographic groups vary in their desire to have children.

Several practical implications of the study may be considered. First, at the personal level, individuals’ awareness of their values may help them better understand their motivations for bringing or not bringing children into the world. Understanding the motivational foundations of childbearing may help individuals better plan their childbearing behavior, e.g., the time of birth and the number of children. In addition, the motivational goals of childbearing may become an important issue in counseling of individuals contemplating birth. At the societal level, understanding changes in value preferences may help to understand or even predict changes in childbirth at the group and country levels. Thus, the study results are practically important at both the individual and societal levels.

## Supporting information

S1 Appendix Path analysis: standardized estimates and *p*-values. (DOCX)

S1 File (SAV)
