# Supplementary material for: The motivational foundations of childbearing: Investigating relationships between childbearing attitudes, value preferences, and parental bonding
Source: PLoS One. 2025 May 29;20(5):e0324243. doi: 10.1371/journal.pone.0324243 (PMC12121918; doi:10.1371/journal.pone.0324243)
Supplement: S1 Appendix — (DOCX) [file pone.0324243.s001.docx]

**Appendix**

**Path Analysis: Standardized Estimates and *p*-Values**

|  | **Women** |  | **Men** |  |
| --- | --- | --- | --- | --- |
| **Effects (Connections between variables in the model)** | **Standardized estimates** | ***p*-values** | **Standardized estimates** | ***p*-values** |
| **Direct effects** |  |  |  |  |
| ***-> Childbearing attitudes*** |  |  |  |  |
| Self-Direction | -.126 | .000 | -.081 | .036 |
| Security Social | .001 | .975 | .125 | .001 |
| Tradition | .231 | .000 | .279 | .000 |
| Benevolence Care | .110 | .000 | .117 | .003 |
| Universalism Nature | -.170 | .000 | -.118 | .004 |
| Maternal bonding | .165 | .000 | .147 | .001 |
| Paternal bonding | .059 | .128 | .092 | .033 |
| Age | -.002 | .965 | -.014 | .754 |
| Education | -.096 | .014 | -.034 | .409 |
| Income | .099 | .008 | .065 | .121 |
| Religiosity | .144 | .001 | .157 | .001 |
| ***-> Self-Direction*** |  |  |  |  |
| Maternal bonding | -.069 | .129 | .069 | .182 |
| Paternal bonding | -.062 | .180 | -.068 | .192 |
| Age | -.096 | .054 | -.062 | .244 |
| Education | .061 | .193 | .145 | .003 |
| Income | -.002 | .973 | -.036 | .477 |
| Religiosity | -.173 | .000 | -.021 | .645 |
| ***-> Security Social*** |  |  |  |  |
| Maternal bonding | -.065 | .163 | .029 | .579 |
| Paternal bonding | .055 | .246 | .008 | .874 |
| Age | .019 | .703 | .027 | .614 |
| Education | .022 | .652 | -.058 | .234 |
| Income | -.024 | .604 | .118 | .019 |
| Religiosity | .036 | .392 | -.018 | .687 |
| ***-> Tradition*** |  |  |  |  |
| Maternal bonding | .090 | .010 | .038 | .362 |
| Paternal bonding | .094 | .008 | .057 | .175 |
| Age | .046 | .228 | .012 | .777 |
| Education | -.096 | .008 | -.083 | .036 |
| Income | .065 | .057 | .035 | .385 |
| Religiosity | .627 | .000 | .572 | .000 |
| ***-> Benevolence Care*** |  |  |  |  |
| Maternal bonding | .121 | .008 | .163 | .001 |
| Paternal bonding | .080 | .086 | -.008 | .881 |
| Age | -.014 | .780 | .011 | .830 |
| Education | .018 | .698 | -.013 | .792 |
| Income | -.021 | .644 | .011 | .819 |
| Religiosity | -.058 | .164 | -.061 | .170 |
| ***-> Universalism Nature*** |  |  |  |  |
| Maternal bonding | -.016 | .729 | .059 | .233 |
| Paternal bonding | -.026 | .575 | -.101 | .042 |
| Age | .080 | .103 | .052 | .312 |
| Education | .007 | .878 | .046 | .333 |
| Income | -.090 | .040 | -.080 | .102 |
| Religiosity | -.214 | .000 | -.253 | .000 |
| ***-> Maternal bonding*** |  |  |  |  |
| Age | -.087 | .084 | -.115 | .30 |
| Education | .063 | .176 | -.028 | .562 |
| Income | .035 | .442 | .065 | .197 |
| Religiosity | .104 | .013 | .037 | .413 |
| ***-> Paternal bonding*** |  |  |  |  |
| Age | -.143 | .004 | -.171 | .001 |
| Education | .188 | .000 | .020 | .678 |
| Income | .032 | .468 | .128 | .101 |
| Religiosity | .109 | .008 | .048 | .284 |
|  |  |  |  |  |
| **Indirect effects** |  |  |  |  |
| ***-> Childbearing attitudes*** |  |  |  |  |
| Age | -.025 | .279 | -.034 | .155 |
| Education | .003 | .879 | -.051 | .019 |
| Income | .039 | .056 | .065 | .004 |
| Religiosity | .230 | .000 | .194 | .000 |
| Maternal bonding | .045 | .008 | .021 | .289 |
| Paternal bonding | .043 | .013 | .033 | .067 |
